# Asymptotic stabilization of underactuated surface vehicles with actuator saturation

**DOI:** 10.7717/peerj-cs.793

**Published:** 2021-11-24

**Authors:** Pengfei Zhang, Tingting Yang

**Affiliations:** Huzhou University, Huzhou, China

**Keywords:** Input saturation, Asymptotically stable, Globally stable, USVs

## Abstract

This paper investigates the problem of global asymptotic stabilization of underactuated surface vessels (USVs) with input saturation. A novel input transformation is presented, so that the USV system can be transformed to a cascade structure. For the obtained system, the improved fractional power control laws are proposed to ensure input signals do not exceed actuator constraints and enhance convergence rates. Finally, stabilization and parameter optimization algorithm of USVs are proposed. Simulations are given to demonstrate the effectiveness of the presented method.

## Introduction

Underactuated surface vehicles (USV) stabilization has many practical uses in engineering practice, such as monitoring, rescue, and fishing. Generally, the USV has no side thruster considered, whose reduction always causes by an actuator failure or a deliberate decision. For example, the limitation of the actuator number due to, *e.g*., cost and weight considerations. This paper establishes a feedback control law to stabilize three states containing both position and orientation with only two available control inputs.

The reference (Pettersen & Egeland, 1996) shows that USVs have no asymptotic stability on desired equilibrium points with time-invariant continuous control laws. The reason is that the dynamic models of USVs have nonholonomic constraints, so they do not meet Brockett’s necessary condition (Brockett & Millman, 1983). Furthermore, the existing control approaches designed for conventional nonholonomic systems cannot stabilize USVs directly since the dynamics of a USV are not drift-less (Oriolo & Nakamura, 1991). For these reasons, significant interest exist in stabilization of USVs as evidenced by various control schemes presented for such a problem (Reyhanoglu, 1996; Pettersen & Egeland, 1996; Ghommam et al., 2006; Ma, 2009; Pettersen & Nijmeijer, 2000; Zhang & Wu, 2014; Mazenc, Pettersen & Nijmeijer, 2002; Dong & Yi, 2005; Ma & Xie, 2013; Zhang & Wu, 2015; Xie & Ma, 2015). To name some, the references (Reyhanoglu, 1996; Pettersen & Egeland, 1996) proposed control laws that can stabilize the system to a small neighborhood of origin. Pettersen & Nijmeijer (2000) proposed a control law guaranteeing the semi-global asymptotic stability of USVs. Ghommam et al. (2006), Ma (2009), Zhang & Wu (2014), Mazenc, Pettersen & Nijmeijer (2002) and Dong & Yi (2005) proposed control methods that can globally asymptotically stabilize USVs. Other related researches on the stabilization control of USVs include, but are not limited to (Ma & Xie, 2013; Zhang & Wu, 2015; Xie & Ma, 2015) and many references therein. To enhance convergence rates (Zhang & Guo, 2018), presents a fast stabilization approach with fractional power terms. Zhang & Guo (2020) proposes the fixed-time control method stabilizing USVs with dead-zones. Further (Guo & Zhang, 2020) considers the case that both yaw constraints and disturbances exist.

This paper is concerned with actuator saturation in the USV stabilization control problem as supplementary studies of the literature (Guo & Zhang, 2020). The above researches have one thing in common: the assumption that there is no limit on the inputs. However, the functional USV movement usually relies on motor propulsion limited by the bouned amount of torque. Thus signals of the control algorithm for USVs should always be constrained. Under such constraints, the stability of the closed system with conventional methods is impacted. Hence, with no processing, the input saturation may affect system accuracy and speed and even cause system instability. Such an issue leads to significant challenges for the USV controller design. In short, developing a stabilization scheme for a USV with input saturation is an arduous task yet has theoretical and practical significance. To this end, the input saturation problem is solved in tracking control of USVs (such as Huang et al. (2015)). However, due to the difference of dynamics between stabilization and tracking, USV stabilization control requires its own anti-input saturation control method. To the best of the authors’ knowledge, possible solutions to the issue of actuator saturation include model predictive control (MPC) (Li & Yan, 2016), which is restricted to the unsolvable or singular point problems.

Motivated by the above discussion, this paper aims to address the stabilization problem of USVs with input saturation. The main contributions of this paper are twofold:

1. For the USVs with input saturation, a novel input transformation method is proposed in this paper. Thus, the USV system with input saturation can be transformed to the cascade structure.
2. An improved fractional power control method is presented to avoid inputs violating actuator saturation constraints.

The organization of this paper is as follows. The 2nd section is to model the USVs and establish the objective. The 3rd section contains input and state transitions so that the USV stabilization problem is solved by stabilizing the obtained two cascaded subsystems. Then the control law of the original USV system is given. This section also provides proof of the global asymptotic stability and the method of parameter optimization. The last section shows the numerical simulations and discussions.

## System modeling and the objective

### Kinematic model

**Figure 1:**
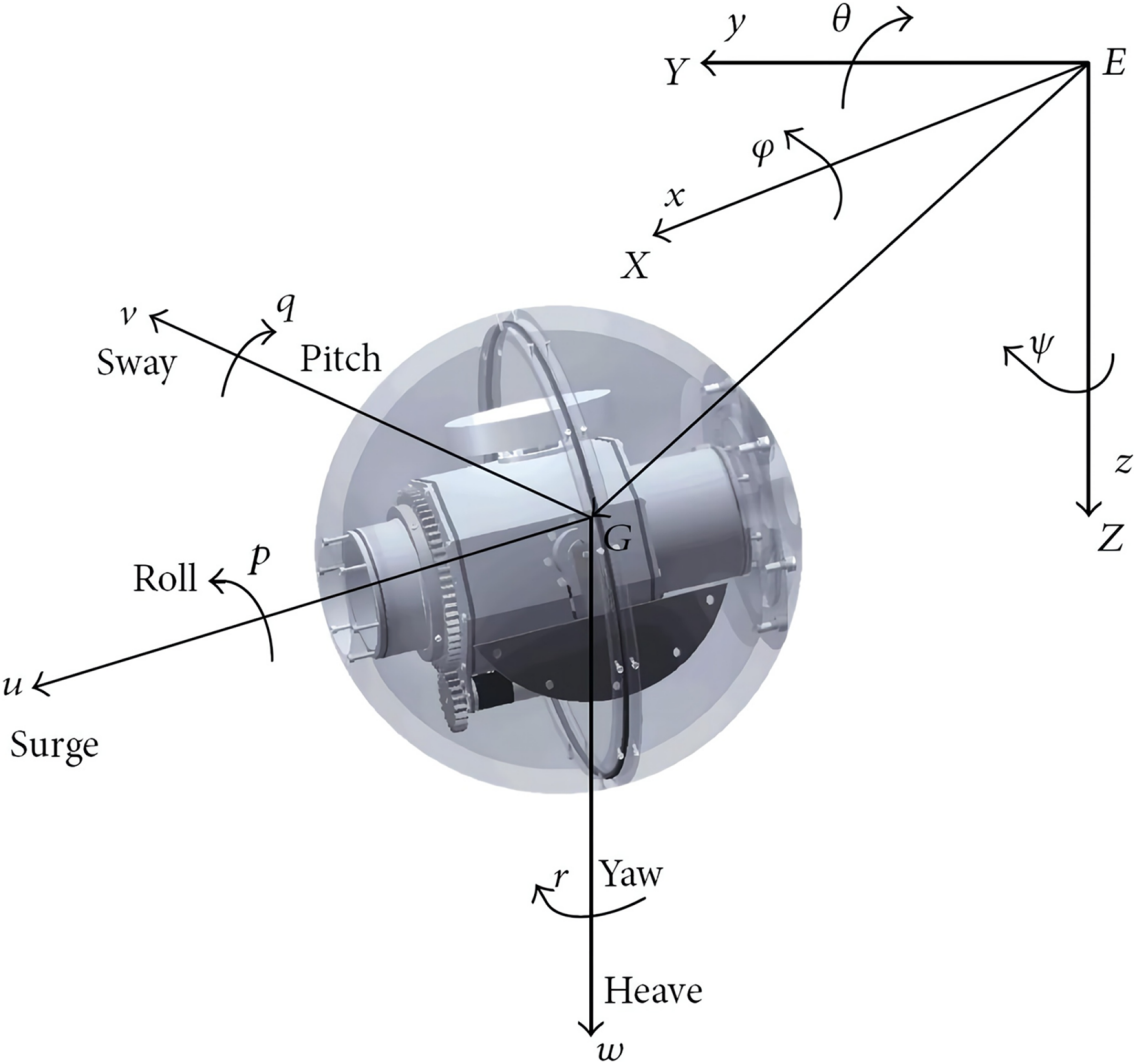
The underactuated surface vehicle.

Consider a USV as depicted in Fig. 1 with an inertial frame {*O*_*G*_} and a body-fixed frame {*O*_*E*_}. Denote *x* and *y* as positions of the USV in an inertial frame, and $\psi$ as the yaw angle relative to the geographic north. The kinematics and of the USV with mismatched and matched disturbances are given as follows:

(1a) $$\left\{ {\matrix{{\dot x = u\cos \psi - v\sin \psi ,} \cr {\dot y = u\sin \psi + v\cos \psi ,} \cr {\dot \psi = r,} \cr } } \right.$$

(1b) $$\left\{ {\matrix{{{\hskip-2pc}\dot u = - \displaystyle{{{d_{11}}} \over {{m_{11}}}}u + \displaystyle{{{m_{22}}} \over {{m_{11}}}}vr + \displaystyle{{{s_{{\tau _{umax}}}}({\tau _u})} \over {{m_{11}}}},} \cr {{\hskip-6pc}\dot v = - \displaystyle{{{d_{22}}} \over {{m_{22}}}}v - \displaystyle{{{m_{11}}} \over {{m_{22}}}}ur,} \cr {\dot r = - \displaystyle{{{d_{33}}} \over {{m_{33}}}}r - \displaystyle{{{m_{22}} - {m_{11}}} \over {{m_{33}}}}uv + \displaystyle{{{s_{{\tau _{rmax}}}}({\tau _r})} \over {{m_{33}}}},} \cr } } \right.$$

where *x*, *y* denotes the coordinate of surface vessel mass center in the earth-fixed frame, *ψ* is the orientation of vessel. Variables *u*, *v*, *r* are the velocities in surge, sway and yaw respectively. Parameters *m*_11_, *m*_22_, *m*_33_ are the inertia coefficients, *d*_11_, *d*_22_, *d*_33_ are the damping coefficients of the vessel. *τ*_*u*_ is the force of surge, *τ*_*r*_ is the torques of yaw. ${m_1} = m - {X_{\dot u}}$, ${m_2} = m - {Y_{\dot v}}$, ${m_3} = m - {N_{\dot r}}$, *d*_1_ = *X*_*u*_, *d*_2_ = *Y*_*v*_, *d*_3_ = *N*_*r*_. ${X_{\dot u}}$, ${Y_{\dot v}}$, ${N_{\dot r}}$, *X*_*u*_, *Y*_*v*_, *N*_*r*_ are the coefficients from Taylor series, which are described in Fossen (1994). The saturation function is defined as follows:

(2) $${s_\beta }(z) = \left\{{\matrix{{z}, & if\quad |z| \le \beta, \cr {\beta \cdot sign(z),} & if\quad |z| > \beta, } } \right.$$

where *β* and *z* denote the saturation constant and arbitrary variable. In terms of Eqs. (1a) and (1b), we can then define that *τ*_*umax*_ and *τ*_*rmax*_ are, respectively, the maximum of input variables *τ*_*u*_ and *τ*_*r*_.

### The objective

The objective of this paper is to design the control inputs *τ*_*u*_ and *τ*_*r*_ that can globally asymptotically stabilize the USV system as modeled in (1a) and (1b) subject to input saturation, namely, the following holds true for any initial conditions $\mathop {\lim }\limits_{t \to + \infty } [x(t),y(t),\psi (t),u(t),v(t),r(t)] = {\bf 0}.$

The saturation restricts the controllbility of USV systems, thus some references (such as Reyhanoglu, 1996, Pettersen & Egeland, 1996, Ghommam et al., 2006, Ma, 2009, Pettersen & Nijmeijer, 2000, Mazenc, Pettersen & Nijmeijer, 2002, Dong & Yi, 2005) are unable to be followed in the presence of saturation making it quite challenging to stabilize system (1a) and (1b) to the origin. As a compensatory research, this paper emphasize the input saturation problem instead of disturbances, input dead-zones and output constraints. This is because the methods in Guo & Zhang (2020) can be directly applied to solve these problems with some saturation constraints. The only work that deals with stabilization of USVs with yaw constraints is Li & Yan (2016). However, it is based on MPC and relies on singular state transformations to ensure iterative feasibility.

## Model transformation

Consider the system (1a) and (1b), similar to the previous literatures about stabilization of USVs, the state transformation is still utilized. However, in terms of the input saturation, existing input transformed methods can not be applied directly. Hence a novel input transformation is proposed in this section under the following assumption.

*Assumption 1*: The parameters *d*_11_, *d*_22_ and *d*_33_ satisfy the following condition: ${({d_{22}} - {d_{11}})^2} < {m_{11}}{d_{11}} \cdot \min \left\{ \displaystyle{{{d_{11}}} \over {{m_{11}}}},\displaystyle{{2{d_{22}}} \over {{m_{22}}}},\displaystyle{{{d_{33}}} \over {{m_{33}}}}\right\} .$

This assumption is relatively strong, and its specific meaning refers to a surface vehicle with a relatively small volume and a slight difference between the horizontal and vertical directions. To facilitate the control design, first the state transformation is cited from Pettersen & Egeland (1996). Define the vector *ϑ* = [*ϑ*_1_, *ϑ*_2_, *ϑ*_3_, *ϑ*_4_, *ϑ*_5_, *ϑ*_6_]^*T*^ with

(3) $$\left\{ {\matrix{ {{\vartheta _1} =  x\cos \psi + y\sin \psi ,} \hfill \cr {{\vartheta _2} =  - x\sin \psi + y\cos \psi + \displaystyle{{{m_{22}}} \over {{d_{22}}}}v,} \hfill \cr {{\vartheta _3} =  \psi, {\vartheta _4} = v,} \hfill \cr {{\vartheta _5} =  - {\vartheta _1} - \displaystyle{{{m_{11}}} \over {{d_{22}}}}u, {\vartheta _6} = r.} \hfill \cr } } \right.$$

According to the system (1a) and (1b), one has

(4a) $$\left\{ {\matrix{ {{{\dot \vartheta }_1} = } \hfill  { - \displaystyle{{{d_{22}}} \over {{m_{11}}}}{\vartheta _1} - \displaystyle{{{d_{22}}} \over {{m_{11}}}}{\vartheta _5} + {\vartheta _2}{\vartheta _6} - \displaystyle{{{m_{22}}} \over {{d_{22}}}}{\vartheta _4}{\vartheta _6},} \hfill \cr {{{\dot \vartheta }_4} = } \hfill  { - \displaystyle{{{d_{22}}} \over {{m_{22}}}}{\vartheta _4} + \displaystyle{{{d_{22}}} \over {{m_{22}}}}{\vartheta _6}({\vartheta _1} + {\vartheta _5}),} \hfill \cr } } \right.$$

(4b) $$\left\{ {\matrix{ {{{\dot \vartheta }_2} = } \hfill  {{\vartheta _5}{\vartheta _6}; {{\dot \vartheta }_3} = {\vartheta _6}} \hfill \cr {{{\dot \vartheta }_5} = } \hfill  {(1 - {d_{11}}d_{22}^{ - 1})u - {\vartheta _2}{\vartheta _6} - d_{22}^{ - 1}{s_{{\tau _{umax}}}}({\tau _u}),} \hfill \cr {{{\dot \vartheta }_6} = } \hfill  { - {d_{33}}m_{33}^{ - 1}{\vartheta _6} - ({m_{11}} - {m_{22}})m_{33}^{ - 1}uv + m_{33}^{ - 1}{s_{{\tau _{rmax}}}}({\tau _r}).} \hfill \cr } } \right.$$

For the transformed system, there are following results cited from previous works:

**Lemma 1** *Stabilization of system (1a) and (1b) is equivalent to that of system (4a) and (4b)*.

**Lemma 2** *If subsystem (4b) is globally asymptotically stable, then systems (4a) and (4b) are globally asymptotically stable*.

According to the Lemmas 1 and 2, the global asymptotic stability of the USV system in (1a) and (1b) can be achieved by designing a global asymptotical control law for subsystem (4b). In addition, for the transformed system (4b), input transformations was usually introduced to further simplify calculations. However, for USVs with saturations, input transformations in previous works (such as Reyhanoglu, 1996; Pettersen & Egeland, 1996; Ghommam et al., 2006; Ma, 2009; Pettersen & Nijmeijer, 2000; Mazenc, Pettersen & Nijmeijer, 2002; Dong & Yi, 2005) cannot be used. Hence, in this paper we propose a novel input transformation for system (4b) to deal with input saturations.

Define parameters *λ*_1_, *λ*_2_, *λ*_3_, *β*_*u*_ and *β*_*r*_ satisfying the following conditions:

(5) $$- \displaystyle{{{m_{11}}} \over {{m_{22}}}}{\lambda _1} + \displaystyle{{{m_{22}}} \over {{m_{11}}}}{\lambda _2} - \displaystyle{{{m_{22}} - {m_{11}}} \over {{m_{33}}}}{\lambda _3} = 0,$$

(6) $${\beta _u} < \min \{ {\tau _{umax}},{\Delta_0},{\Delta_1},{\Delta_2}\} ,$$

(7) $${\varepsilon _1} - {\varepsilon _2} < {\beta _r} < \min \{ {\gamma _1}{\beta _u},{\varepsilon _1} + {\varepsilon _2},{\tau _{rmax}}\} ,$$

where constant parameters *γ*_1_, *γ*_2_, *γ*_3_, *ε*_1_, *ε*_2_, *Δ*_0_, *Δ*_1_, *Δ*_2_ and *Δ*_3_ are defined as:

}{}{\gamma _1} = \sqrt {\displaystyle{{{m_{33}}{d_{33}}{\lambda _1}{\gamma _3}} \over {{\lambda _3}{{({d_{22}} - {d_{11}})}^2}}}\left[1 - \displaystyle{{{{({d_{22}} - {d_{11}})}^2}} \over {{m_{11}}{d_{11}}{\gamma _3}}}\right]} ,

\eqalign{ {\gamma _2} = \displaystyle{{2({m_{11}} - {m_{22}})} \over {\sqrt {{\lambda _1}{\lambda _2}} }},\ {\gamma _3} = \min \left\{ \displaystyle{{{d_{11}}} \over {{m_{11}}}},\displaystyle{{2{d_{22}}} \over {{m_{22}}}},\displaystyle{{{d_{33}}} \over {{m_{33}}}}\right\},\ {\varepsilon _1} = \displaystyle{{{m_{33}}{d_{33}}{\gamma _3}} \over {{\lambda _3}{\gamma _2}}}, \cr  {\varepsilon _2} = \sqrt {\varepsilon _1^2 - \displaystyle{{{\lambda _1}{m_{33}}{d_{33}}\beta _u^2} \over {{\lambda _3}{m_{11}}{d_{11}}}}},\ {\Delta_0} = \displaystyle{{{\gamma _3}} \over {{\gamma _2}}}\sqrt {\displaystyle{{{m_{11}}{m_{33}}{d_{11}}{d_{33}}} \over {{\lambda _1}{\lambda _3}}}},\ {\Delta_1} = \displaystyle{{2{\gamma _1}{\gamma _3}{m_{33}}{d_{33}}} \over {{\lambda _3}{\gamma _2}\left(\gamma _1^2 + \displaystyle{{{\lambda _1}{m_{33}}{d_{33}}} \over {{\lambda _3}{m_{11}}{d_{11}}}}\right)}}, \cr  {\Delta_2} = \sqrt {\displaystyle{{{\lambda _3}{m_{11}}{d_{11}}} \over {{\lambda _1}{m_{33}}{d_{33}}}}\left[\varepsilon _1^2 - \displaystyle{{sign({\Delta_3}) + 1} \over 2}\Delta_3^2\right]},\ {\Delta_3} = {\varepsilon _1} - {\tau _{rmax}}.}

According to the Assumption 1 and inequality (6), we can have $1 - \displaystyle{{{{({d_{22}} - {d_{11}})}^2}} \over {{m_{11}}{d_{11}}{\gamma _3}}} > 0, \varepsilon _1^2 - \displaystyle{{{\lambda _1}{m_{33}}{d_{33}}\beta _u^2} \over {{\lambda _3}{m_{11}}{d_{11}}}} > 0,{\varepsilon _1} - {\varepsilon _2} < {\gamma _1}{\beta _u}, {\varepsilon _1} - {\varepsilon _2} < {\tau _{rmax}}.$ Above inequalities indicate that *γ*_1_ and *ε*_2_ are real and the inequality (7) is hold. Then for system (4b), we have the following result:

**Lemma 3** *For the USV system (1a) and (1b), if we choose*

(8) $$\matrix{ {{\tau _u} = {s_{{\beta _u}}}[({d_{22}} - {d_{11}})u - {d_{22}}{s_{{\beta _1}}}({\varpi _1})],{\tau _r} = {s_{{\beta _r}}}[({m_{11}} - {m_{22}})uv + {m_{33}}{s_{{\beta _2}}}({\varpi _2})].} \hfill \cr }$$

there must be positive constants *β*_1_ and *β*_2_ satisfying that ${\beta _1} \lt \displaystyle{{{\beta _u}} \over {{d_{22}}}} - \displaystyle{{({d_{22}} - {d_{11}})|u|} \over {{d_{22}}}},  {\beta _2} \lt \displaystyle{{{\beta _r}} \over {{m_{33}}}} - \displaystyle{{({m_{11}} - {m_{22}})|uv|} \over {{m_{33}}}},$ where *β*_*u*_ < *τ*_*umax*_ and *β*_*r*_ < *τ*_*rmax*_ are positive constants, *ϖ*_1_ and *ϖ*_2_ are virtual inputs.

*Proof:* Define the Lyapunov function as ${V_0} = \displaystyle{{{\lambda _1}} \over 2}{u^2} + \displaystyle{{{\lambda _2}} \over 2}{v^2} + \displaystyle{{{\lambda _3}} \over 2}{r^2},$ whose derivative is ${\dot V_0} = - \displaystyle{{{d_{11}}} \over {{m_{11}}}}{\lambda _1}{u^2} - \displaystyle{{{d_{22}}} \over {{m_{22}}}}{\lambda _2}{v^2} - \displaystyle{{{d_{33}}} \over {{m_{33}}}}{\lambda _3}{r^2} + \displaystyle{{{\tau _u}} \over {{m_{11}}}}{\lambda _1}u + \displaystyle{{{\tau _r}} \over {{m_{22}}}}{\lambda _2}r.$ Together with the fact that |*τ*_*u*_| ≤ *β*_*u*_ and |*τ*_*r*_| ≤ *β*_*r*_,

(9) $${\dot V_0} \le - {\gamma _3}{V_0} + \displaystyle{{{\lambda _1}\beta _u^2} \over {2{m_{11}}{d_{11}}}} + \displaystyle{{{\lambda _3}\beta _r^2} \over {2{m_{33}}{d_{33}}}}.$$

According to the inequality in (9), we can have

(10) $$u \le \sqrt {\displaystyle{{2\beta } \over {{\lambda _1}}}}, v \le \sqrt {\displaystyle{{2\beta } \over {{\lambda _2}}}}, r \le \sqrt {\displaystyle{{2\beta } \over {{\lambda _3}}}} .$$

where $\beta = \displaystyle{{{\lambda _1}\beta _u^2} \over {2{m_{11}}{d_{11}}{\gamma _3}}} + \displaystyle{{{\lambda _3}\beta _r^2} \over {2{m_{33}}{d_{33}}{\gamma _3}}}$. According to the Eq. (6), we can have ${\beta _r} < {\gamma _1}{\beta _u}, \displaystyle{{{m_{33}}{d_{33}}{\gamma _3}} \over {{\lambda _3}{\gamma _2}}} - \varepsilon < {\beta _r} < \displaystyle{{{m_{33}}{d_{33}}{\gamma _3}} \over {{\lambda _3}{\gamma _2}}} + \varepsilon .$ The above inequality implies $[1 - \displaystyle{{{{({d_{22}} - {d_{11}})}^2}} \over {{m_{11}}{d_{11}}{\gamma _3}}}]\beta _u^2 > \displaystyle{{{\lambda _3}{{({d_{22}} - {d_{11}})}^2}} \over {{\lambda _1}{m_{33}}{d_{33}}{\gamma _3}}}\beta _r^2$ and ${\beta _r} > {\gamma _2}\left(\displaystyle{{{\lambda _1}\beta _u^2} \over {2{m_{11}}{d_{11}}{\gamma _3}}} + \displaystyle{{{\lambda _3}\beta _r^2} \over {2{m_{33}}{d_{33}}{\gamma _3}}}\right).$ This means that $|(d_{22}-d_{11})u{|}^{2}\leq |d_{22}-d_{11}{|}^{2}\frac{2\beta }{\lambda_{1}}=|d_{22}-d_{11}{|}^{2}\left(\frac{\beta_{u}^{2}}{m_{11}d_{11}\gamma_{3}}+\frac{\lambda_{3}\beta_{r}^{2}}{\lambda_{1}m_{33}d_{33}\gamma_{3}}\right)<\beta_{u}^{2},$ and $|(m_{11}-m_{22})uv|\leq |(m_{11}-m_{22})|\frac{2\beta }{\lambda_{1}\lambda_{2}}=\frac{2(m_{11}-m_{22})}{\sqrt{\lambda_{2}}}\left(\frac{\beta_{u}^{2}}{2m_{11}d_{11}\gamma_{3}}+\frac{\lambda_{3}\beta_{r}^{2}}{2m_{33}d_{33}\gamma_{3}}\right)<\beta_{r}.$ Therefore, there must be constants *β*_1_ and *β*_2_ satisfying that ${\beta _1} < \displaystyle{{{\beta _u}} \over {{d_{22}}}} - \displaystyle{{|({d_{22}} - {d_{11}})u|} \over {{d_{22}}}},{\beta _2} < \displaystyle{{{\beta _r}} \over {{m_{33}}}} - \displaystyle{{|({m_{11}} - {m_{22}})uv|} \over {{m_{33}}}}.$ Hence we can have *β*_*u*_ > |*d*_22_ − *d*_11_|·|*u*| + *d*_22_|*β*_1_| and *β*_*r*_ > |*m*_11_ − *m*_22_|·|*uv*| + *m*_33_|*β*_2_|. The proof is closed.

According to the Lemma 3, the facts *β*_*u*_ < *τ*_*umax*_ and *β*_*r*_ < *τ*_*rmax*_, finally, we can have the following input transformation:

(11) $$\begin{array}{c} \tau_{u}=(d_{22}-d_{11})u-d_{22}s_{\beta_{1}}(\varpi_{1})\leq \beta_{u}<\tau_{umax},\\ \tau_{r}=(m_{11}-m_{22})uv+m_{33}s_{\beta_{2}}(\varpi_{2})\leq \beta_{r}<\tau_{rmax}. \end{array}$$

Since *τ*_*u*_ < *τ*_*umax*_ and *τ*_*r*_ < *τ*_*rmax*_, we can have,

(12) $${s_{{\tau _u}max}}({\tau _u}) = {\tau _u}, {s_{{\tau _r}max}}({\tau _r}) = {\tau _r}.$$

Then the system (4b) can be transformed to the following cascade system:

(13) $$\matrix{ {{{\dot \vartheta }_2} = } \hfill & {{\vartheta _5}{\vartheta _6}, {{\dot \vartheta }_3} = {\vartheta _6},} \hfill & {} \hfill \cr {{{\dot \vartheta }_5} = } \hfill & { - {\vartheta _2}{\vartheta _6} + {s_{{\beta _1}}}({\varpi _1}), {{\dot \vartheta }_6} = } \hfill & { - {\gamma _4}{\vartheta _6} + {s_{{\beta _2}}}({\varpi _2}).} \hfill \cr }$$

where ${\gamma _4} = \displaystyle{{{d_{33}}} \over {{m_{33}}}}$.

**Remark 1** *By the proposed input transformation, the system (4b) who has input saturations can be further simplified as (13). This is one innovation point of this paper*.

**Remark 2** *According to the inequalities (6) and (7), the values of β*_*u*_ *and β*_*r*_ *depend on the terms* $\displaystyle{{{\gamma _3}} \over {{\gamma _2}}}\sqrt {\displaystyle{{{m_{11}}{m_{33}}{d_{11}}{d_{33}}} \over {{\lambda _1}{\lambda _3}}}}$, Δ_1_, Δ_2_, $\displaystyle{{{m_{33}}{d_{33}}{\gamma _3}} \over {{\lambda _3}{\gamma _2}}} + \varepsilon$ *and γ*_1_. *According to definitions of these parameters, we can have*

\eqalign{{\Delta_0} = {\gamma _3}\sqrt {\displaystyle{{{m_{11}}{m_{33}}{d_{11}}{d_{33}}} \over {2({m_{11}} - {m_{22}})}}} \sqrt {\displaystyle{{{\lambda _2}} \over {{\lambda _3}}}} ,{\Delta_1} = \displaystyle{{({d_{22}} - {d_{11}})} \over {\sqrt {({m_{11}} - {m_{22}})} }}\sqrt {\Delta_1^*} \sqrt {\displaystyle{{{\lambda _2}} \over {{\lambda _3}}}}, \displaystyle{{{m_{33}}{d_{33}}{\gamma _3}} \over {{\lambda _3}{\gamma _2}}} \cr\quad+ \varepsilon \ge \displaystyle{{{m_{33}}{d_{33}}{\gamma _3}} \over {{m_{22}} - {m_{11}}}}\displaystyle{{\sqrt {{\lambda _1}{\lambda _2}} } \over {{\lambda _3}}}, {\gamma _1}{\beta _u} = {\beta _u}\sqrt {\displaystyle{{{m_{33}}{d_{33}}{\gamma _3}} \over {{{({d_{22}} - {d_{11}})}^2}}}\left[1 - \displaystyle{{{{({d_{22}} - {d_{11}})}^2}} \over {{m_{11}}{d_{11}}{\gamma _3}}}\right]} \sqrt {\displaystyle{{{\lambda _1}} \over {{\lambda _3}}}}, }

*with $\Delta_1^* = {m_{33}}{d_{33}}{\gamma _3}\left[1 - \displaystyle{{{{({d_{22}} - {d_{11}})}^2}} \over {{m_{11}}{d_{11}}{\gamma _3}}}\right]$. Obviously, since $- \displaystyle{{{m_{11}}} \over {{m_{22}}}}{\lambda _1} + \displaystyle{{{m_{22}}} \over {{m_{11}}}}{\lambda _2} - \displaystyle{{{m_{22}} - {m_{11}}} \over {{m_{33}}}}{\lambda _3} = 0$, similar positive values of ${m_{11}}m_{22}^{ - 1}{\lambda _1}$ and ${m_{22}}m_{11}^{ - 1}{\lambda _2}$ can result in smaller *λ*_3_ and larger Δ_1_, Δ_2_ and $\displaystyle{{{m_{33}}{d_{33}}{\gamma _3}} \over {{\lambda _3}{\gamma _2}}} + \varepsilon$. However, this implies that $\displaystyle{{{m_{33}}{d_{33}}{\gamma _3}\sqrt {{\lambda _1}{\lambda _2}} } \over {{\lambda _3}({m_{11}} - {m_{22}})}} \gt\gt {\tau _{rmax}}$. Hence we can have ${\Delta_2} \approx \sqrt {\displaystyle{{{m_{11}}{d_{11}}{\tau _{rmax}}{\gamma _3}} \over {{m_{11}} - {m_{22}}}}} {\left(\displaystyle{{{\lambda _2}} \over {{\lambda _1}}}\right)^{\textstyle{1 \over 4}}}.$ A large $\displaystyle{{{\lambda _2}} \over {{\lambda _1}}}$ is useful to the large Δ_2_ and contradictory with the requirement of similar ${m_{11}}m_{22}^{ - 1}{\lambda _1}$ and ${m_{22}}m_{11}^{ - 1}{\lambda _2}$. Therefore, the difficulty lies that there exists a trade-off in selecting these parameters *λ*_1_, *λ*_2_ and *λ*_3_. The details are stated in Section Optimization*.

## Stabilization of usvs

Although the system is simplified as (13), there still be input saturation problem in the transformed system implying that the existing methodologies for (13) are not applicable here. To overcome the challenge, in this section, we propose a novel control law by combining the control law in Section “Stabilization of USVS” with the proposed input transformation in the Model Transformation section to solve the stabilization problem of the underactuated surface vessel.

### Control of transformed system

Define *β*_3_, *β*_4_, *β*_5_, *β*_6_ and *β*_7_ as arbitrary constants satisfying that

(14) $$\beta_{4}+\beta^{\frac{1}{2}}\lambda_{3}^{-\frac{1}{2}}\beta_{3}<\beta_{1},\beta_{5}+\beta_{6}+\beta_{7}<\beta_{2}.$$

where *λ*_3_, *β*_1_ and *β*_2_ are defined in (5) and (8). We cite the Barbalat Lemma which the results in this paper will base on:

**Lemma 4** *Slotine & Li (1991) Let* $z:R \to R$ *be a uniformly continuous function on *[0,∞)*. Suppose that* $\mathop {\lim }\limits_{t \to \infty } \int_0^t z(s)ds$ *exists and is finite. Then*, $\mathop {\lim }\limits_{t \to \infty } z(t) = 0$.

Based on the above parameters, we can have the following control law for system (13):

(15) $$\matrix{ {{\varpi _1} = } \hfill & { - {\beta _3}\arctan [{\kappa _1}{\vartheta _2}]{\vartheta _6} - {\beta _4}\arctan [{\kappa _2}{\vartheta _5}],} \hfill \cr {{\varpi _2} = } \hfill & { - {\beta _5}\arctan ({\kappa _3}{\vartheta _3}) - {\beta _6}\arctan ({\kappa _4}{\vartheta _6}) + {\beta _7}si{g^\xi }\{ \arctan [{\kappa _1}{\vartheta _2}]\} \rho (t).} \hfill \cr }$$

Here, $si{g^\xi }\left\{ {\arctan [{\kappa _1}{\vartheta _2}]} \right\} = sign\left\{ {\arctan [{\kappa _1}{\vartheta _2}]} \right\} \cdot |\arctan [{\kappa _1}{\vartheta _2}{]|^\xi }$, *κ*_1_, *κ*_2_, *κ*_3_ and *κ*_4_ are positive constants, *ρ*(*t*) = sin *t*, $\xi = \displaystyle{p \over q}$, where *p* is positive odd and *q* is positive even. According to the Eq. (14), we can have $\varpi_{1}\leq \beta_{3}\cdot \sqrt{\frac{\lambda_{1}\beta_{u}^{2}}{\lambda_{3}m_{11}d_{11}\gamma_{3}}+\frac{\beta_{r}^{2}}{m_{33}d_{33}\gamma_{3}}}+\beta_{4}<\beta_{1}$ and $\varpi_{2}\leq \beta_{5}+\beta_{6}+\beta_{7}<\beta_{2}.$ This implies that the transformed inputs *τ*_*u*_ and *τ*_*r*_ can be less that their saturation values *β*_*u*_ and *β*_*r*_. Then substituting controller (15) into the system in (13), we can have

(16) $$\matrix{ {{{\dot \vartheta }_2} = } \hfill & {{\vartheta _5}{\vartheta _6}, {{\dot \vartheta }_3} = {\vartheta _6},} \hfill \cr {{{\dot \vartheta }_5} = } \hfill & { - {\vartheta _2}{\vartheta _6} - {\beta _3}\arctan [{\kappa _1}{\vartheta _2}]{\vartheta _6} - {\beta _4}\arctan [{\kappa _2}{\vartheta _5}],} \hfill \cr {{{\dot \vartheta }_6} = } \hfill & { - {\gamma _4}{\vartheta _6} - {\beta _5}\arctan ({\kappa _3}{\vartheta _3}) - {\beta _6}\arctan ({\kappa _4}{\vartheta _6}) + {\beta _7}si{g^\xi }\left\{ {\arctan [{\kappa _1}{\vartheta _2}]} \right\}\rho (t).} \hfill \cr }$$

For the system (16), we have the following result:

**Lemma 5** *The system (16) is globally asymptotically stable*.

*Proof*: Consider the following Lyapunov function:

(17) $$\matrix{ {{V_1} = \displaystyle{1 \over 2}\vartheta _2^2 + {\beta _3}\arctan ({\kappa _1}{\vartheta _2}){\vartheta _2} - \displaystyle{{{\beta _3}} \over {2{\kappa _1}}}\ln (1 + \kappa _1^2\vartheta _2^2) + \displaystyle{1 \over 2}\vartheta _5^2,} \hfill \cr }$$

whose derivative satisfies that ${\dot V_1} = - {\beta _4}\arctan ({\vartheta _5}){\vartheta _5} \le 0.$ The Lyapunov function *V*_1_ is monotonically decreasing for *ϑ*_5_ ≠ 0 and bounded, there must be a minimum value of *V*_1_. Since *ϑ*_2_ and *ϑ*_5_ are bounded and ${\dot \vartheta _3} = {\vartheta _6},{\dot \vartheta _6} = - {\gamma _4}{\vartheta _6} - {\beta _5}\arctan ({\kappa _3}{\vartheta _3}) - {\beta _6}\arctan ({\kappa _4}{\vartheta _6}) + {\beta _7}si{g^\xi }\{ \arctan [{\kappa _1}{\vartheta _2}]\} \rho (t)$, states *ϑ*_3_ and *ϑ*_6_ are bounded. Take the derivative of ${\dot V_1}$, ${\ddot V_1} = - {\beta _4}[\arctan ({\vartheta _5}) + \displaystyle{{{\vartheta _5}} \over {1 + \vartheta _5^2}}][ - {\vartheta _2}{\vartheta _6} - {\beta _3}\arctan ({\kappa _1}{\vartheta _2}){\vartheta _6} - {\beta _4}\arctan ({\kappa _2}{\vartheta _5})].$ Since states *ϑ*_2_ and *ϑ*_5_ are bounded, then ${\dot V_1}$ and ${\ddot V_1}$ are continuous and bounded. This implies that ${\dot V_1}$ is uniformly continuous, according to the Lemma 4, we can have that $\mathop {\lim }\limits_{t \to + \infty } {\vartheta _5} = 0$. Define *Γ*_1_ = *ϑ*_5_, according to Appendix A, ${\dot \Gamma _1}$ is uniformly continuous. Together with Lemma 4, we have

(18) $$\matrix{ {\mathop {\lim }\limits_{t \to + \infty } {{\dot \Gamma }_1} = \mathop {\lim }\limits_{t \to + \infty } [ - {\vartheta _2}{\vartheta _6} - {\beta _3}\arctan ({\kappa _1}{\vartheta _2}){\vartheta _6} - {\beta _4}\arctan ({\kappa _2}{\vartheta _5})] = 0.} \hfill \cr }$$

Since $\mathop {\lim }\limits_{t \to + \infty } {\vartheta _5} = 0$ and states *ϑ*_2_ and *ϑ*_6_ are bounded, the Eq. (18) indicates that $\mathop {\lim }\limits_{t \to + \infty } \arctan ({\kappa _1}{\vartheta _2}){\vartheta _6} = 0.$ Due to *ϑ*_2_ is bounded, the above equation indicates that there must be a positive real number *K* = *ξ* + 2 satisfying that

(19) $$\matrix{ {\mathop {\lim }\limits_{t \to + \infty } {{[\arctan ({\kappa _1}{\vartheta _2})]}^{K - \xi }}{\vartheta _6} = \mathop {\lim }\limits_{t \to + \infty } {{[\arctan ({\kappa _1}{\vartheta _2})]}^{K - \xi - 1}}\arctan ({\kappa _1}{\vartheta _2}){\vartheta _6} = 0.} \hfill \cr }$$

Define Γ_2_ = [arctan(*κ*_1_*ϑ*_2_)]^*K*^^−^^*ξ*^*ϑ*_6_, according to Appendix B, ${\dot \Gamma _2}$ is uniformly continuous, according to Lemma 4, we can then have

(20) $$\eqalign{{\mathop {\lim }\limits_{t \to + \infty } {{\dot \Gamma }_2} = \mathop {\lim }\limits_{t \to + \infty } (K - \xi )[\arctan ({\kappa _1}{\vartheta _2}{{)]}^{K - \xi - 1}}\displaystyle{{{\kappa _1}{\vartheta _5}\vartheta _6^2} \over {1 + \kappa _1^2\vartheta _2^2}}  + {{[\arctan ({\kappa _1}{\vartheta _2})]}^{K - \xi }}}  \cr\quad {[ - {\gamma _4}{\vartheta _6} - }  {{\beta _5}\arctan ({\kappa _3}{\vartheta _3}) - {\beta _6}\arctan ({\kappa _4}{\vartheta _6}) + {\beta _7}si{g^\xi }\{ \arctan [{\kappa _1}{\vartheta _2}]\} \rho (t)].}}$$

Due to the fact that $\mathop {\lim }\limits_{t \to + \infty } {\vartheta _5} = 0$, $\mathop {\lim }\limits_{t \to + \infty } {[\arctan ({\kappa _1}{\vartheta _2})]^{K - \xi }}{\vartheta _6} = 0$ and *ϑ*_3_, *ϑ*_2_, *ϑ*_6_ are bounded. The Eq. (20) means that $\mathop {\lim }\limits_{t \to + \infty } ({\kappa _3}{\beta _5}\arctan ({\kappa _3}{\vartheta _3})[\arctan ({\kappa _1}{\vartheta _2}{)]^{K - \xi }} - {\beta _7}{[\arctan ({\kappa _1}{\vartheta _2})]^K}\rho (t)) = 0.$

Define Γ_3_ = *κ*_3_*β*_5_arctan(*κ*_3_*ϑ*_3_)[arctan(*κ*_1_*ϑ*_2_)]^*K*^^−^^*ξ*^ − *β*_7_*ρ*(*t*) ×[arctan(*κ*_1_*ϑ*_2_)]^*K*^, according to the Appendix C, we can have that ${\dot \Gamma _3}$ is uniformly continuous, and according to Lemma 4,

$\mathop {\lim }\limits_{t \to + \infty } {\dot \Gamma _3}(t) = \mathop {\lim }\limits_{t \to + \infty } \displaystyle{{\kappa _3^2{\beta _5}{\vartheta _6}} \over {1 + \kappa _3^2\vartheta _3^2}}{[\arctan ({\kappa _1}{\vartheta _2})]^{K - \xi }} + \displaystyle{{{\kappa _1}{\kappa _3}{\beta _5}{\vartheta _5}{\vartheta _6}} \over {1 + \kappa _1^2\vartheta _2^2}}K\arctan ({\kappa _3}{\vartheta _3})$ $\times {[\arctan ({\kappa _1}{\vartheta _2})]^{K - \xi - 1}} - \displaystyle{{{\kappa _1}{\kappa _3}{\beta _5}{\vartheta _5}{\vartheta _6}} \over {1 + \kappa _1^2\vartheta _2^2}}\xi \arctan ({\kappa _3}{\vartheta _3})[\arctan ({\kappa _1}{\vartheta _2}{)]^{K - \xi - 1}}$

$- {\beta _7}K{[\arctan ({\kappa _1}{\vartheta _2})]^{K - 1}}\displaystyle{{{\kappa _1}{\vartheta _5}{\vartheta _6}} \over {1 + \kappa _1^2\vartheta _2^2}} - {\beta _7}{[\arctan ({\kappa _1}{\vartheta _2})]^K}\dot \rho (t).$

Together with the fact that $\mathop {\lim }\limits_{t \to + \infty } {\vartheta _5} = 0$, $\mathop {\lim }\limits_{t \to + \infty } {[\arctan ({\kappa _1}{\vartheta _2})]^{K - \xi }}$ ×*ϑ*_6_ = 0 and *ϑ*_3_, *ϑ*_2_, *ϑ*_6_ are bounded, we can have $\mathop {\lim }\limits_{t \to + \infty } {\beta _7}\dot \rho (t)$ $\times {[\arctan ({\kappa _1}{\vartheta _2})]^K} = 0.$ Obviously, the state *ϑ*_2_ converges to zero, according to (18), dynamics of *ϑ*_3_ and *ϑ*_6_ can be ${\dot \vartheta _3} = {\vartheta _6},{\dot \vartheta _6} =  - {\gamma _4}{\vartheta _6} - {\beta _5}\arctan ({\kappa _3}{\vartheta _3}) - {\beta _6}\arctan ({\kappa _4}{\vartheta _6}).$

Define ${V_2} = {\beta _5}\arctan ({\kappa _3}{\vartheta _3}){\vartheta _3} - \displaystyle{{{\beta _5}} \over {2{\kappa _3}}}\ln (1 + \kappa _3^2\vartheta _3^2) \ge 0,$ whose derivative satisfies ${\dot V_2} = - {\gamma _4}\vartheta _6^2 - {\beta _6}\arctan ({\kappa _4}{\vartheta _6}){\vartheta _6} \le 0.$ Therefore, *ϑ*_3_ and *ϑ*_6_ can all converge to zero globally and asymptotically. The proof is closed.

### The control algorithm for USVs

Based on the above discussions, we are now in a position to come back to the USV system in (1a) and (1b) and give the following controller,

(21a) $$\matrix{ {{\tau _u} = } { - {d_{22}}{s_{{\beta _1}}}( - {\beta _3}\arctan [{\kappa _1}{\vartheta _2}]{\vartheta _6} - {\beta _4}\arctan [{\kappa _2}{\vartheta _5}]) + ({d_{22}} - {d_{11}})u,} \hfill \cr }$$

(21b) $$\eqalign{ {\tau _r} = {({m_{11}} - {m_{22}})uv + [ - {\beta _5}\arctan ({\kappa _3}{\vartheta _3}) - {\beta _6}\arctan ({\kappa _4}{\vartheta _6})} \cr\quad + {\beta _7}si{g^\xi } \{ \arctan [{\kappa _1}{\vartheta _2}]\} \times  {m_{33}}{s_{{\beta _2}}}\rho (t)],}$$

with ${\beta _1} < {\beta _u}d_{22}^{ - 1} - |({d_{22}} - {d_{11}})u|d_{22}^{ - 1}$, ${\beta _2} < {\beta _r}m_{33}^{ - 1} - |({m_{11}} - {m_{22}})uv|m_{33}^{ - 1}$, $\beta_{4}+\beta^{\frac{1}{2}}\lambda_{3}^{-\frac{1}{2}}\beta_{3}<\beta_{1}$, *β*_5_ + *β*_6_ + *β*_7_<*β*_2_. Here *β*_*r*_, *β*_*r*_ and *λ*_3_ are constants defined in the Eqs. (5)–(7).

We now have the following theorem, whose proof can be done along similar lines of Lemmas 1, 2 and 5.

**Theorem 1** *For input saturation USV system (1a) and (1b), the fractional power controller (21a) and (21b) ensure states x, y, ψ, u, v, and r globally asymptotically converged to zero*.

Next, to facilitate practical application and summarize the main results, we provide the following algorithms to implement the proposed methods and results in USV.

### Stabilization control algorithm of USVs with input saturation

**Step 1**. Take measurements *τ*_*umax*_, *τ*_*rmax*_, *m*_11_, *m*_22_, *m*_33_, *d*_11_, *d*_22_ and *d*_33_ of the USV.

**Step 2**. Let $\varepsilon = \min \left\{ \displaystyle{{{d_{11}}} \over {{m_{11}}}},\displaystyle{{2{d_{22}}} \over {{m_{22}}}},\displaystyle{{{d_{33}}} \over {{m_{33}}}}\right\}$ and ${\gamma _4} = \displaystyle{{{d_{33}}} \over {{m_{33}}}}$.

**Step 3**. The parameters *λ*_1_, *λ*_2_, *λ*_3_, *β*_*u*_ and *β*_*r*_ are chosen as the Eqs. (5)–(7) and (24).

**Step 4**. Make state transformation *ϑ*_1_, *ϑ*_2_, *ϑ*_3_, *ϑ*_4_, *ϑ*_5_ *ϑ*_6_.

\eqalign{ {\vartheta _1} = xcos\psi + ysin\psi ,{\vartheta _2} = - xsin\psi + ycos\psi + \displaystyle{{{m_{22}}} \over {{d_{22}}}}v,{\vartheta _3} = \psi, \cr\quad {\vartheta _4} = v,{\vartheta _5} = - {\vartheta _1} - \displaystyle{{{m_{11}}} \over {{d_{22}}}}u,{\vartheta _6} = r.

**Step 5**. Let *κ*_1_, *κ*_2_ are arbitrary positive constants, *κ*_3_ = *β*_5_*ε*_1_, *κ*_4_ = *β*_5_*ε*_1_, *ξ* = *p*/*q*, where *p* is positive odd and *q* is positive even.

**Step 6**. Compute virtual control inputs ${\varpi_1} and ${\varpi_2}

${\varpi _1} = - {\beta _3}si{g^\xi }\left\{ {\tanh [{\kappa _1}{\vartheta _2}]} \right\}{\vartheta _6} - {\beta _4}\tanh [{\kappa _2}{\vartheta _5}],\; {\varpi _2} = - {s_{{\beta _5}}}({\kappa _3}{\vartheta _3}) + {\beta _7}si{g^{{\xi _2}}}\left\{ {\tanh [{\kappa _1}{\vartheta _2}]} \right\}\rho (t) - {s_{{\beta _6}}}({\kappa _4}{\vartheta _6}).$

**Step 7**. Compute real control inputs *τ*_*u*_ and *τ*_*r*_ as ${\tau _u} = ({d_2} - {d_1})u - {d_2}{s_{{\beta _1}}}({\varpi _1}), {\tau _r} = ({m_1} - {m_2})uv + {m_3}{s_{{\beta _2}}}({\varpi _2})$.

### Parameter optimization

In this subsection, the optimization algorithm of parameters *λ*_1_, *λ*_2_ and *λ*_3_ for the stabilization of the USV subject to input constraints are introduced.

To facilitate the calculation, first, define positive constant *s* as *λ*_1_ = *sλ*_2_ satisfying $0 < s < \displaystyle{{{b_1}} \over {{b_2}}}$. Then we can have

(22) $${\lambda _3} = ({b_1} - {b_2}s){\lambda _2},$$

where ${b_1} = \displaystyle{{{m_{22}}{m_{33}}} \over {({m_{22}} - {m_{11}}){m_{11}}}}$, ${b_2} = \displaystyle{{{m_{11}}{m_{33}}} \over {({m_{22}} - {m_{11}}){m_{22}}}}$ are positive constants. According to the Remark 2, when *s* satisfies the case that $0 < \sqrt s \le \textstyle{{\sqrt {T_4^2 + 4{b_1}{b_2}\tau _{rmax}^2} - {T_4}} \over {2{b_2}{\tau _{rmax}}}},$ Δ_0_ and Δ_1_ can be small. Therefore, we consider only the case that

(23) $$\sqrt s > \displaystyle{{\sqrt {T_4^2 + 4{b_1}{b_2}\tau _{rmax}^2} - {T_4}} \over {2{b_2}{\tau _{rmax}}}}.$$

According to Eqs. (5)–(7), we have ${\Delta_0} = \textstyle{{{T_1}} \over {\sqrt {{b_1} - {b_2}s} }},{\Delta_1} = \textstyle{{{T_2}} \over {\sqrt {{b_1} - {b_2}s} }},  {\Delta_2} = \textstyle{{{T_3}} \over {\sqrt s }}\sqrt {{T_4}\sqrt s - ({b_1} - {b_2}s){\tau _{rmax}}} ,$ where ${T_1} = {\gamma _3}\sqrt {\textstyle{{{m_{11}}{m_{33}}{d_{11}}{d_{33}}} \over {2({m_{33}} - {m_{11}})}}}$, ${T_2} = \textstyle{{{d_{22}} - {d_{11}}} \over {{m_{11}} - {m_{22}}}}\sqrt {{m_{33}}{d_{33}}{\gamma _3}[1 - \textstyle{{{{({d_{22}} - {d_{11}})}^2}} \over {{m_{11}}{d_{11}}{\gamma _3}}}]}$, ${T_3} = \sqrt {\textstyle{{{m_{11}}{d_{11}}{\tau _{rmax}}} \over {{m_{33}}{d_{33}}}}}$ and ${T_4} = \textstyle{{{m_{33}}{d_{33}}{\gamma _3}} \over {({m_{11}} - {m_{22}})}}$ are constants.

Therefore, the partial derivatives of Δ_0_, Δ_1_ and Δ_2_ about *s* can be $\textstyle{{\partial {\Delta_0}} \over {\partial s}} = \textstyle{{{b_2}{T_1}} \over {2({b_1} - {b_2}s{)^{\textstyle{3 \over 2}}}}} \gt 0,\textstyle{{\partial {\Delta_1}} \over {\partial s}} = \textstyle{{{b_2}{T_2}} \over {2({b_1} - {b_2}s{)^{\textstyle{3 \over 2}}}}} \gt 0,\textstyle{{\partial {\Delta_2}} \over {\partial s}} = \textstyle{{{T_3}[ - \textstyle{{{T_4}\sqrt s } \over 2} + {b_1}{\tau _{rmax}}]} \over {2{s^2}\sqrt {{T_4}\sqrt s - ({b_1} - {b_2}s){\tau _{rmax}}} }}$. This means that Δ_0_ and Δ_1_ are increasing about *s* and the sign of $\textstyle{{\partial {\Delta_2}} \over {\partial s}}$ is determined by the values of *s* and *τ*_*rmax*_. Next, relations of $\textstyle{{\partial {\Delta_2}} \over {\partial s}}$ and the values of *s* and *τ*_*rmax*_ will be analyzed as different cases.

According to the formula (23), it is easy to know that $\frac{2b_{1}b_{2}\tau_{rmax}^{2}-T_{4}\sqrt{T_{4}^{2}+4b_{1}b_{2}\tau_{rmax}^{2}}}{2b_{2}^{2}\tau_{rmax}^{2}}<s<\frac{b_{1}}{b_{2}}.$ Define ${\Delta_4} = - \textstyle{{{T_4}\sqrt s } \over 2} + {b_1}{\tau _{rmax}}$, we can have ${b_1}{\tau _{rmax}} - \textstyle{{{T_4}\sqrt {{b_1}} } \over {2\sqrt {{b_2}} }} < {\Delta_4} < \Delta_4^*,$ $\Delta_4^* = ({b_1} + \textstyle{{2{b_1}{b_2}\tau _{rmax}^2 - {T_4}\sqrt {T_4^2 + 4{b_1}{b_2}\tau _{rmax}^2} } \over {2{b_2}\tau _{rmax}^2}})\textstyle{{{\tau _{rmax}}} \over 2}.$

Case 1: If ${\tau _{rmax}} \ge \textstyle{{{T_4}} \over {2\sqrt {{b_1}{b_2}} }}$, then we can have Δ_4_ > 0 and $\textstyle{{\partial {\Delta_2}} \over {\partial s}} > 0$ meaning Δ_0_, Δ_1_ and Δ_2_ are increasing. Therefore, in order to obtain a large *β*_*u*_, we choose $s = \textstyle{{{b_1}} \over {{b_2}}} - \kappa$, where *κ* is the small positive constant.

Case 2: If ${\tau _{rmax}} < \textstyle{{{T_4}} \over {2\sqrt {{b_1}{b_2}} }}$, according to the value of s, the case can be divided into two intervals.

Case 2.1: When the parameter *s* satisfies that $\frac{2b_{1}b_{2}\tau_{rmax}^{2}-T_{4}\sqrt{T_{4}^{2}+4b_{1}b_{2}\tau_{rmax}^{2}}}{2b_{2}^{2}\tau_{rmax}^{2}}<s\leq \frac{4b_{1}^{2}}{T_{4}^{2}}\tau_{rmax}^{2},$ we can have Δ_4_ >0 and $\textstyle{{\partial {\Delta_2}} \over {\partial s}} \gt 0$ meaning Δ_0_, Δ_1_ and Δ_2_ are increasing. Therefore, in order to obtain a large *β*_*u*_, we choose $s = \textstyle{{4b_1^2} \over {T_4^2}}\tau _{rmax}^2$.

Case 2.2: When $\textstyle{{4b_1^2} \over {T_4^2}}\tau _{rmax}^2 < s < \textstyle{{{b_1}} \over {{b_2}}}$, we can have Δ_4_ < 0. This indicates terms Δ_0_ and Δ_1_ are increasing, Δ_2_ is decreasing about *s*. Hence, if the values of min{Δ_0_,Δ_1_} and Δ_2_ are more approximative, the term min{Δ_0_,Δ_1_,Δ_2_} can be smaller. Define the index function *Q* as $Q = \textstyle{1 \over 2}[(\min \{ {\Delta_0},{\Delta_1}{\} )^2} - \Delta_2^2] = \textstyle{1 \over 2}\Delta_5^2,$ where ${\Delta_5} = \textstyle{{T_5^2} \over {{b_1} - {b_2}s}} - T_3^2\left[\textstyle{{{T_4}} \over {\sqrt s }} - \left(\textstyle{{{b_1}} \over s} - {b_2}\right){\tau _{rmax}}\right]$ and *T*_5_ = min{*T*_1_, *T*_2_}. Then the partial derivative of *Q* about *s* is $\textstyle{{\partial Q} \over {\partial s}} = {\Delta_5} \cdot {\Delta_6},$ where ${\Delta_6} = \textstyle{{\partial {\Delta_5}} \over {\partial s}} = \textstyle{{{b_2}{T_5}} \over {{{({b_1} - {b_2}s)}^2}}} + \textstyle{{T_3^2} \over {{s^2}}}\left(\textstyle{{{T_4}} \over 2}\sqrt s - {b_1}{\tau _{rmax}}\right) > 0$. Since $\textstyle{{\partial {\Delta_5}} \over {\partial s}} = {\Delta_6} > 0$, then Δ_5_ is increasing. Generally, *T*_2_ < *T*_1_ and *T*_5_ = *T*_2_, then we can have ${\Delta_5} = \textstyle{{T_3^2} \over {s({b_1} - {b_2}s)}}{\Delta_7},$ where ${\Delta_7} = b_2^2{\tau _{rmax}}{s^2} + {T_4}{b_2}{s^{\textstyle{3 \over 2}}} + {T_6}s - {T_4}{b_1}\sqrt s + b_1^2{\tau _{rmax}}$, ${T_6} = \textstyle{{T_5^2} \over {T_3^2}} - 2{b_1}{b_2}{\tau _{rmax}}$. This means $\mathop {\lim }\limits_{s \to {b_1}b_2^{ - 1}} {\Delta_5}(s) = + \infty > 0.$ Thus the minimum value of *Q* is determined by different cases of the values of Δ_5_ for $s = \textstyle{{4b_1^2} \over {T_4^2}}\tau _{rmax}^2$.

Case 2.2.1: If Δ_5_ < 0 for $s = \textstyle{{4b_1^2} \over {T_4^2}}\tau _{rmax}^2$, there must exist *s* = s* subject to $\frac{4b_{1}^{2}}{T_{4}^{2}}\tau_{rmax}^{2}<s^{*}<\frac{b_{1}}{b_{2}}$ and $b_2^2{\tau _{rmax}}{s^*}2 + {T_4}{b_2}{s^{*\textstyle{3 \over 2}}} + {T_6}{s^*} - {T_4}{b_1}\sqrt {{s^*}} + b_1^2{\tau _{rmax}} = 0.$ If *s* > s*, then $\textstyle{{\partial {J_1}} \over {\partial s}} > 0$, similarly, if *s*< s* then $\textstyle{{\partial {J_1}} \over {\partial s}} < 0$. This implies that *J*_1_(*s* = s*) is the minimum of *J*_1_. Then, we choose *s* = s*.

Case 2.2.2: If Δ_5_ ≥ 0 for $s = \textstyle{{4b_1^2} \over {T_4^2}}\tau _{rmax}^2$, we can have Δ_5_ > 0. This indicates that $\textstyle{{\partial {J_1}} \over {\partial s}} > 0$, *J*_1_ is increasing. In order to obtain the minimum of *J*_1_, we choose $s = \textstyle{{4b_1^2} \over {T_4^2}}\tau _{rmax}^2$.

Finally, summarizing above conclusions, $\textstyle{{{\lambda _1}} \over {{\lambda _2}}} = s$ can be chosen as:

(24) $$s=\{\begin{array}{ll} \frac{b_{1}}{b_{2}}-\kappa , & if\tau_{rmax}\geq \frac{T_{4}}{2\sqrt{b_{1}b_{2}}},\\ s^{*}or\frac{4b_{1}^{2}}{T_{4}^{2}}\tau_{rmax}^{2}, & elseif\Delta_{5}\left(s=\frac{4b_{1}^{2}}{T_{4}^{2}}\tau_{rmax}^{2}\right)<0,\\ \frac{4b_{1}^{2}}{T_{4}^{2}}\tau_{rmax}^{2}, & elseif\Delta_{5}\left(s=\frac{4b_{1}^{2}}{T_{4}^{2}}\tau_{rmax}^{2}\right)\geq 0. \end{array}$$

**Remark 3** *In this subsection, we analyze the selection method of parameters λ*_1_, *λ*_2_ *and λ*_3_. *In fact, generally*, ${\tau _{rmax}} \ge \textstyle{{{T_4}} \over {2\sqrt {{b_1}{b_2}} }}$. *Thus in most practical cases, we choose* $s = \textstyle{{{b_1}} \over {{b_2}}} - \kappa$.

## Numerical simulations

Numerical simulations are given to illustrate the effectiveness of the presented method. In this section, let the USV have the following initial conditions: [*x*(0), *y*(0), *ψ*(0), *u* (0), *v*(0), *r*(0)]^*T*^ = [3, −3,0,0,0,0]^*T*^. The controller parameters in (21a) and 21b, the inertial matrix and damping matrix in the USV model are given as Huang et al. (2015): *m*_11_ = 200 *kg*, *m*_22_ = 250 *kg*, *m*_33_ = 50 *kg*, *d*_11_ = 70 *kg*/*s*, *d*_22_ = 100 *kg*/*s*, *d*_33_ = 70 *kg*/*s*. The maximum value of the absolute value of control input *τ*_*u*_ and *τ*_*r*_ are *τ*_*umax*_ = 200 *N* and *τ*_*rmax*_ = 200 *N*.

According to the Eq. (22), we can have that *b*_1_ = 1.25, *b*_2_ = 0.8, *T*_1_ = 207.0628, *T*_2_ = 26.7261, *T*_3_ = 74.8331 and *T*_4_ = 17.5. From the inequality (24), we choose *s* = 1.562. Substituting it into the Eq. (23), we can have Δ_0_ = 1035.3, Δ_1_ = 1,336.3 and Δ_2_ = 277.45. This means *β*_*u*_ satisfies that

}{}{\beta _u} < \min \{ 1035.3,\ 1336.3,\ 277.45,\ 500\} .

In this paper, we choose *β*_*u*_ = 250. According to above results and formulas (7) and (2), one has $809.037 < {\beta _r} < \min \{ 6220.2,5387,1000\} .$ We choose *τ*_*r*_ = *τ*_*rmax*_ = 1,000. Thus together with Lemma 3, we can have *β*_1_ < 1.4163, *β*_2_ < 3.6929 and choose *β*_1_ = 1.4 and *β*_2_ = 3.6. Finally, according to formulas (14), we choose *β*_3_ = 0.0018, *β*_4_ = 1, *β*_5_ = 1, *β*_6_ = 0.6, *β*_7_ = 2, *κ*_1_ = 10,000, *κ*_2_ = 1, *κ*_3_ = 1 and *κ*_4_ = 1.

**Figure 2:**
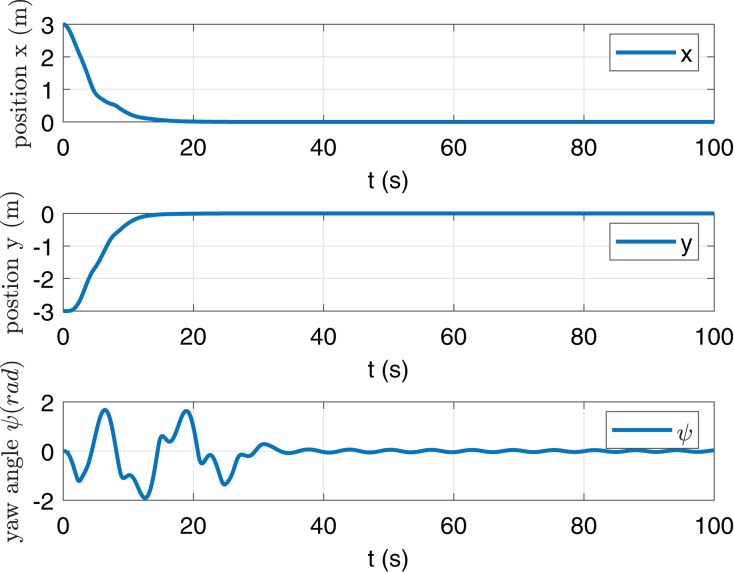
State trajectories *x*, *y* and *ψ*.

**Figure 3:**
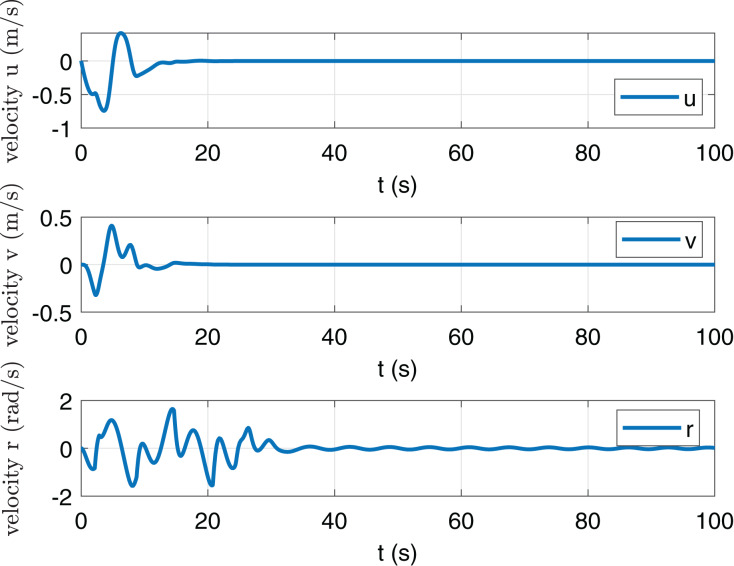
State trajectories *u*, *v* and *r*.

**Figure 4:**
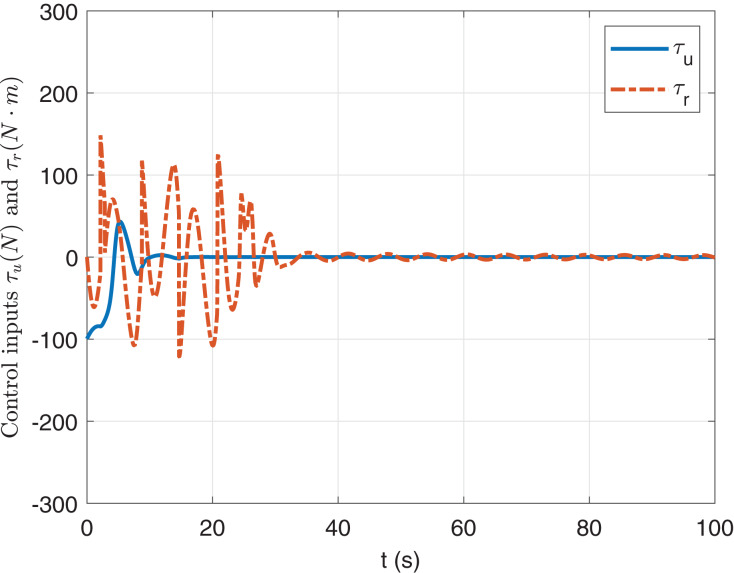
Input trajectories *τ*_u_ and *τ*_r_.

In Figs. 2 and 3, it is shown that states *x*, *y*, *ψ*, *u*, *v* and *r* can be converged to zero. Fig. 4 shows the torque *τ*_*u*_ and *τ*_*r*_. It is obvious that the maximum value of |*τ*_*u*_| and |*τ*_*r*_| are less than *τ*_*umax*_ and *τ*_*rmax*_ respectively.

As is known, there must be some trade-off in the input and convergence of states. In this paper, we consider the input saturation of USVs, meaning that the performance may be reduced. To investigate the performance of our controller with other methods, to be specific, consider the following performance index

(25) $$Q = {x^2} + {y^2} + {\psi ^2} + {u^2} + {v^2} + {r^2}.$$

**Figure 5:**
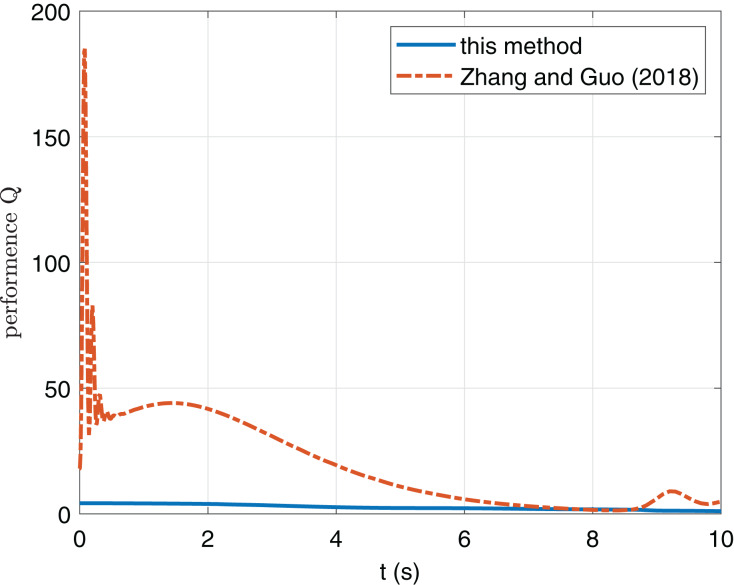
Comparison of performance index Q of this paper and Zhang & Guo (2018).

Figure 5 shows that compared with the method in Zhang & Guo (2018), the convergence time is increased in this paper, which is caused by the input saturation term.

**Figure 6:**
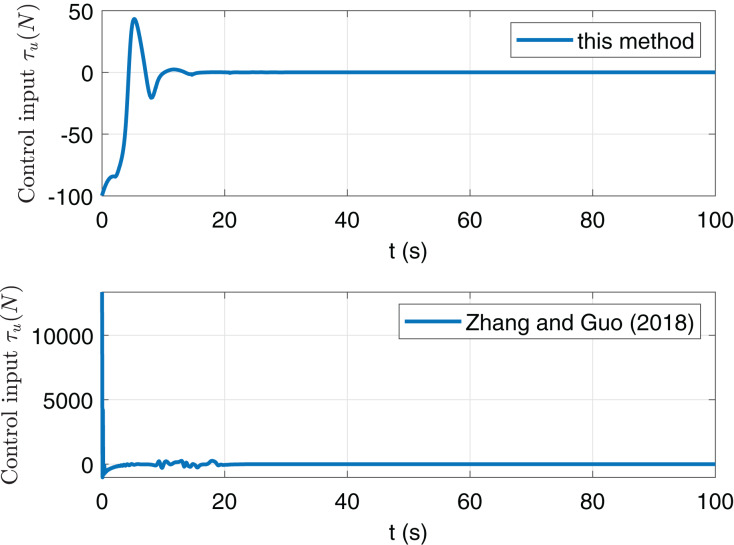
Comparison of input *τ*_u_ of this paper and Zhang & Guo (2018).

**Figure 7:**
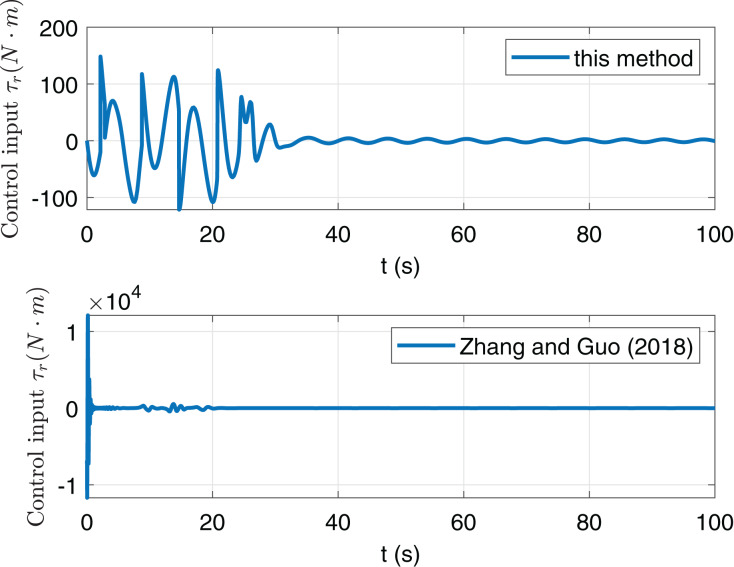
Comparison of consumption index *J* of this paper and Zhang & Guo (2018).

However, in Figs. 6 and 7, the maximum of *τ*_*u*_ and *τ*_*r*_ in this paper are far less than them in paper Zhang & Guo (2018). This implies that the anti-saturation control law in this paper is at work and obviously reduces the burden of actuators. Thus we now focus on the comparison of consumption. Consider the following consumption index

(26) $$J = \int_0^t \tau _u^2(s) + \tau _r^2(s)ds.$$

**Figure 8:**
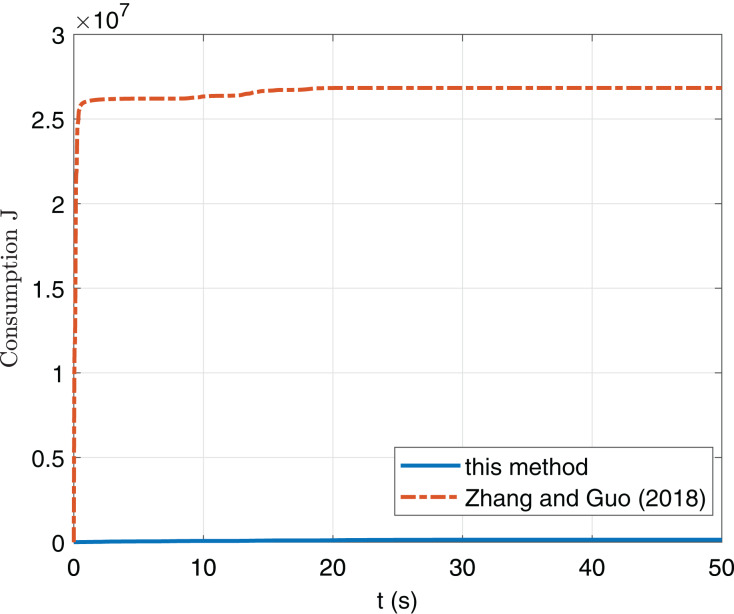
Comparison of consumption index *J* of this paper and Zhang & Guo (2018).

We show the energy cost in Fig. 8, from which we can see that the energy consumption is far less than that of Zhang & Guo (2018).

**Figure 9:**
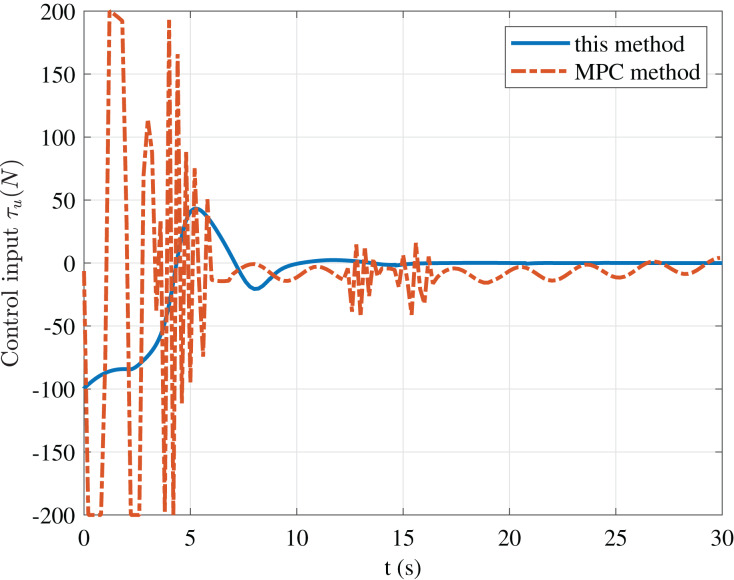
Comparison of control input *τ*_u_ of this paper and MPC method.

**Figure 10:**
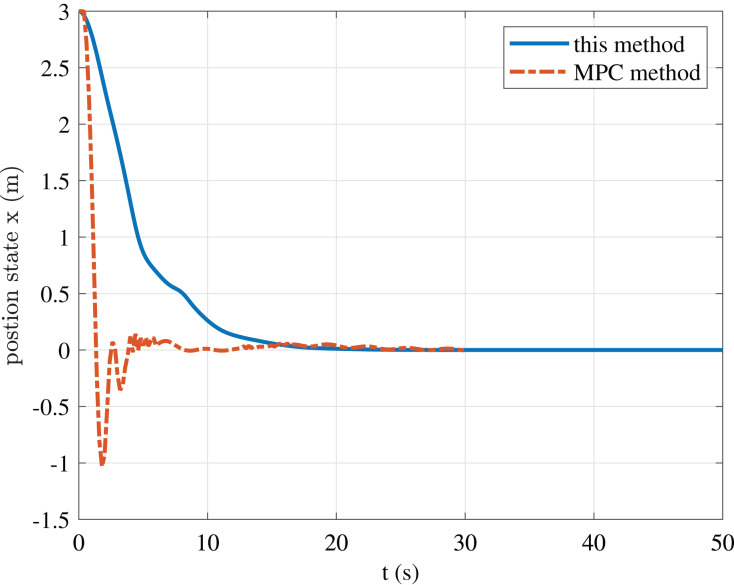
Comparison of position state *x* of this paper and the MPC method.

**Figure 11:**
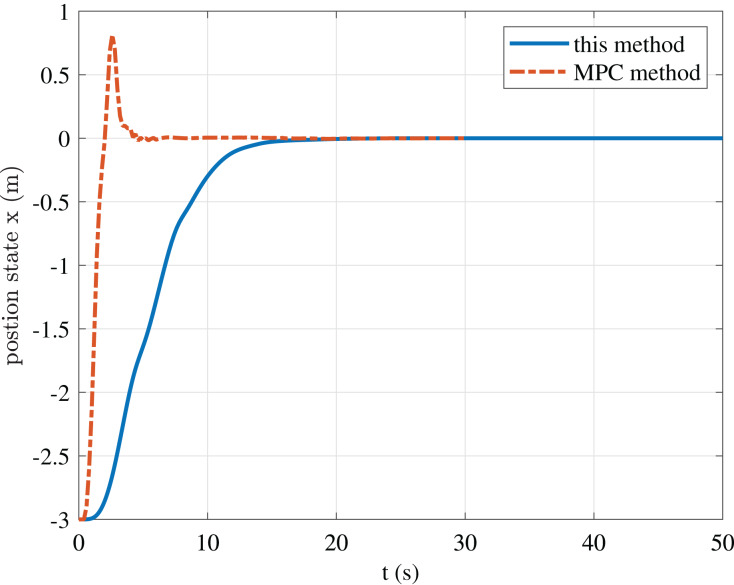
Comparison of position state *y* of this paper and the MPC method.

To further illustrate the advantages of the method in this paper, we give a comparison result with the MPC casadi-windows-matlabR2016a-v3.4.5 method in Figs. 9–11.

The figure shows that although the MPC method can obtain faster convergence performance than our method. However, its control input oscillation frequency is relatively high, which requires relatively high actuators. Thus, we do not emphasize that our approach is superior to the MPC method, but proposes a particular strategy for USV stabilization control to avoid the problems of high oscillation frequency, difficulty in solving, and increased requirements for computing power in the MPC method.

## Conclusion and future work

In this paper, by introducing a novel input, an improved transformation is proposed. For the transformed system, a fractional power control law is presented to realize the global asymptotic stability of the USV with input saturation, and a parameter optimization method is given.

The output limitation caused by the environment and the task may have a meaningful impact on the transient behavior and even the system’s stability. The stability of the output-constrained USV is still an open issue. The energy consumption optimization and stability control of USV is another interesting problem for future research. At the same time, due to the extreme randomness of the environment faced by the sea task, some control methods for stochastic nonlinear systems, such as Zhang & Wang (2021), Yin et al. (2019), Zhang et al. (2016), Zhang, Hu & Gow (2020), should also be considered for application in the USV stabilization control in the future.

## Appendix

### Appendix A

Then, the proof of uniform continuity of ${\dot \Gamma _1}$ is shown. Take the 1st and 2nd derivative of Γ_1_, we can have ${\dot \Gamma _1} = - {\vartheta _2}{\vartheta _6} - {\beta _3}\arctan ({\kappa _1}{\vartheta _2}){\vartheta _6} - {\beta _4}\arctan ({\kappa _2}{\vartheta _5}),$ ${{\ddot \Gamma }_1} = {\vartheta _2}{\vartheta _5}{\vartheta _6} - {\vartheta _2}[ - {d_{33}}m_{33}^{ - 1}{\vartheta _6} - ({m_{11}} - {m_{22}})m_{33}^{ - 1}uv + m_{33}^{ - 1}{s_{{\tau _{rmax}}}}({\tau _r})] - \displaystyle{{{\beta _3}{\vartheta _5}\vartheta _6^2} \over {1 + \kappa _1^2\vartheta _2^2}}+ \displaystyle{{{\kappa _2}{\beta _4}{\vartheta _2}{\vartheta _6}} \over {1 + \kappa _2^2\vartheta _5^2}}$ $+ \displaystyle{{{\kappa _2}{\beta _4}\{ {\beta _3}\arctan [{\kappa _1}{\vartheta _2}]{\vartheta _6} + {\beta _4}\arctan [{\kappa _2}{\vartheta _5}]\} } \over {1 + \kappa _2^2\vartheta _5^2}}{\beta _3}\arctan ({\kappa _1}{\vartheta _2})$ $\times [ - {\gamma _4}{\vartheta _6} - {\beta _5}\arctan ({\kappa _3}{\vartheta _3}) - {\beta _6}\arctan ({\kappa _4}{\vartheta _6}) + {\beta _7}si{g^\xi }\{ \arctan [{\kappa _1}{\vartheta _2}]\} \rho (t)].$

Due to the fact that states *ϑ*_2_, *ϑ*_3_, *ϑ*_5_ and *ϑ*_6_ are bounded, according to above equations, ${\dot \Gamma _2}$ and ${\ddot \Gamma _2}$ are continuous and bounded. This implies ${\dot \Gamma _1}$ is uniformly continuous.

### Appendix B

Here, the proof of uniform continuity of ${\dot \Gamma _2}$ is shown. Take the 1st and 2nd derivative of Γ_2_, we can have ${{\dot \Gamma }_2} = (K - \xi )[\arctan ({\kappa _1}{\vartheta _2}{)]^{K - \xi - 1}}\displaystyle{{{\kappa _1}{\vartheta _5}\vartheta _6^2} \over {1 + \kappa _1^2\vartheta _2^2}} + {[\arctan ({\kappa _1}{\vartheta _2})]^{K - \xi }}[ - {\gamma _4}{\vartheta _6} - {\beta _5}\arctan ({\kappa _3}{\vartheta _3}) - {\beta _6}\arctan ({\kappa _4}{\vartheta _6}) + {\beta _7}si{g^\xi }\{ \arctan [{\kappa _1}{\vartheta _2}]\} \rho (t)],$ ${{\ddot \Gamma }_2} = (K - \xi )[\arctan ({\kappa _1}{\vartheta _2}{)]^{K - \xi - 1}}\displaystyle{{{\kappa _1}{\vartheta _5}\vartheta _6^2} \over {1 + \kappa _1^2\vartheta _2^2}}[ - {\gamma _4}{\vartheta _6} - {\beta _5}\arctan ({\kappa _3}{\vartheta _3}) - {\beta _6}\arctan ({\kappa _4}{\vartheta _6}) + {\beta _7}si{g^\xi }\{ \arctan [{\kappa _1}{\vartheta _2}]\} \rho (t)] + (K - \xi )(K - \xi - 1) {[\arctan ({\kappa _1}{\vartheta _2})]^{K - \xi - 2}} \displaystyle{{\kappa _1^2\vartheta _5^2\vartheta _6^4} \over {{{(1 + \kappa _1^2\vartheta _2^2)}^2}}} +$ $ [\arctan(\kappa_1\vartheta_2)]^{K-\xi-1}{\frac\displaystyle{K-\xi} \over {\displaystyle(1+\kappa_1^2\vartheta_2^2)^2}}\{\kappa_1[-\vartheta_2\vartheta_6 -{\beta_{3}}\arctan(\kappa_1\vartheta_2)\vartheta_6-{\beta_{4}}\arctan(\kappa_2\vartheta_5)]  \vartheta_6^2 +2\kappa_1\vartheta_5[-\gamma_4\vartheta_6-\beta_{5}\arctan(\kappa_3\vartheta_3)-\beta_{6}\arctan(\kappa_4\vartheta_6) +\beta_{7}sig^{\xi}\{\arctan[\kappa_1\vartheta_2]\}\rho(t)] $ $ -2\kappa_1^2\vartheta_2\vartheta_5\vartheta_6\} + [\arctan(\kappa_1\vartheta_2)]^{K-\xi}\{-\gamma_4[-\gamma_4\vartheta_6-\beta_{5}\arctan(\kappa_3\vartheta_3)  -\beta_{6}\arctan(\kappa_4\vartheta_6)  + \beta_{7}sig^{\xi}\{\arctan[\kappa_1\vartheta_2]\}\rho(t)] -{\frac\displaystyle{\kappa_3\beta_{5}\vartheta_6} \over \displaystyle{1+\kappa_3^2\vartheta_3^2}} $[-{\frac\displaystyle{\kappa_4\beta_{6} \big[-\gamma_4\vartheta_6-\beta_{5}\arctan(\kappa_3\vartheta_3) \beta_{6}\arctan(\kappa_4\vartheta_6) - \beta_{7}sig^{\xi}\left\{\arctan\left[\kappa_1\vartheta_2\right]\right\}\rho(t)\big]} \over \displaystyle{1+\kappa_4^2\vartheta_6^2}} $ $+\beta_{7}sig^{K}\dot{\rho}(t)\times\left\{\arctan\left[\kappa_1\vartheta_2\right]\right\} +\beta_{7}Ksig^{K-1}\left\{\arctan\left[\kappa_1\vartheta_2\right]\right\}{\frac\displaystyle{\kappa_1\vartheta_5\vartheta_6} \over \displaystyle{1+\kappa_1^2\vartheta_2^2}}.$

Due to states *ϑ*_2_, *ϑ*_3_, *ϑ*_5_ and *ϑ*_6_ are bounded and continuous. ${\dot \Gamma _2}$ and ${\ddot \Gamma _2}$ are also bounded and continuous. Hence ${\dot \Gamma _2}$ is uniformly continuous.

### Appendix C

At last, the proof of uniform continuity of ${\dot \Gamma _3}$ is shown. Take the 1st and 2nd derivative of Γ_3_, we can have ${\dot \Gamma _3} = \displaystyle{{\kappa _3^2{\beta _5}{\vartheta _6}} \over {1 + \kappa _3^2\vartheta _3^2}}A_2^{K - \xi }$ $+ \displaystyle{{{\kappa _1}{\kappa _3}{\beta _5}{\vartheta _5}{\vartheta _6}} \over {1 + \kappa _1^2\vartheta _2^2}}K\arctan ({\kappa _3}{\vartheta _3})A_2^{K - \xi - 1}$ $- \displaystyle{{{\kappa _1}{\kappa _3}{\beta _5}{\vartheta _5}{\vartheta _6}} \over {1 + \kappa _1^2\vartheta _2^2}}\xi \arctan ({\kappa _3}{\vartheta _3})A_2^{K - \xi - 1} -$ ${\beta _7}KA_2^{K - 1}\displaystyle{{{\kappa _1}{\vartheta _5}{\vartheta _6}} \over {1 + \kappa _1^2\vartheta _2^2}} - {\beta _7}A_2^K\dot \rho (t),$ ${\ddot \Gamma _3} = (K - \xi )\displaystyle{{\kappa _3^2{\beta _5}{\vartheta _6}} \over {1 + \kappa _3^2\vartheta _3^2}}\displaystyle{{{\kappa _1}{\vartheta _5}{\vartheta _6}} \over {1 + \kappa _1^2\vartheta _2^2}}A_2^{K - \xi - 1} + \displaystyle{{\kappa _3^2{\beta _5}} \over {1 + \kappa _3^2\vartheta _3^2}}A_2^{K - \xi }$ $[ - {\gamma _4}{\vartheta _6} - {\beta _5} \times \arctan ({\kappa _3}{\vartheta _3}) - {\beta _6}\arctan ({\kappa _4}{\vartheta _6})$ $+ {\beta _7}si{g^\xi }\left\{ {{A_2}} \right\}\rho (t)] - \displaystyle{{2\kappa _3^2{\beta _5}} \over {1 + \kappa _3^2\vartheta _3^2}}$ $\times A_2^{K - \xi }\kappa _3^2{\vartheta _3}{\vartheta _6}$ $+ \displaystyle{{{\kappa _1}{\kappa _3}{\beta _5}{\vartheta _5}{\vartheta _6}} \over {1 + \kappa _1^2\vartheta _2^2}}({K^2} - K\xi - K)\arctan ({\kappa _3}{\vartheta _3})A_2^{K - \xi - 2}$ $+ K\displaystyle{{{\kappa _3}{\vartheta _6}} \over {1 + \kappa _3^2\vartheta _3^2}}\displaystyle{{{\kappa _1}{\kappa _3}{\beta _5}{\vartheta _5}{\vartheta _6}} \over {1 + \kappa _1^2\vartheta _2^2}}A_2^{K - \xi - 1}$ $+ \displaystyle{{{\kappa _1}{\kappa _3}{\beta _5}} \over {{{(1 + \kappa _1^2\vartheta _2^2)}^2}}}K{\dot \vartheta _5}{\vartheta _6}\arctan ({\kappa _3}{\vartheta _3})A_2^{K - \xi - 1}$ $+ \displaystyle{{{\kappa _1}{\kappa _3}{\beta _5}} \over {{{(1 + \kappa _1^2\vartheta _2^2)}^2}}}K\arctan ({\kappa _3}{\vartheta _3})A_2^{K - \xi - 1} \times {\vartheta _5}{\dot \vartheta _6}- 2\displaystyle{{{\kappa _1}{\kappa _3}{\beta _5}} \over {{{(1 + \kappa _1^2\vartheta _2^2)}^2}}}K\arctan ({\kappa _3}{\vartheta _3})A_2^{K - \xi - 1} $ $\times \kappa _1^2{\vartheta _2}{\vartheta _5}{\vartheta _6} - \displaystyle{{{\kappa _1}{\kappa _3}{\beta _5}{\vartheta _5}{\vartheta _6}} \over {1 + \kappa _1^2\vartheta _2^2}}  (K\xi - {\xi ^2} - 1)\arctan ({\kappa _3}{\vartheta _3})A_2^{K - \xi - 2}$ $- \xi \displaystyle{{{\kappa _3}{\vartheta _6}} \over {1 + \kappa _3^2\vartheta _3^2}}\displaystyle{{{\kappa _1}{\kappa _3}{\beta _5}{\vartheta _5}{\vartheta _6}} \over {1 + \kappa _1^2\vartheta _2^2}}A_2^{K - \xi - 1}$ $- \displaystyle{{{\kappa _1}{\kappa _3}{\beta _5}} \over {{{(1 + \kappa _1^2\vartheta _2^2)}^2}}}\xi \arctan ({\kappa _3}{\vartheta _3})A_2^{K - \xi - 1}{\dot \vartheta _5}{\vartheta _6}$ $- \displaystyle{{{\kappa _1}{\kappa _3}{\beta _5}} \over {{{(1 + \kappa _1^2\vartheta _2^2)}^2}}}\xi \arctan ({\kappa _3}{\vartheta _3})A_2^{K - \xi - 1} \times  {\vartheta _5}{\dot \vartheta _6 + 2\displaystyle{{{\kappa _1}{\kappa _3}{\beta _5}} \over {{{(1 + \kappa _1^2\vartheta _2^2)}^2}}}\xi \arctan ({\kappa _3}{\vartheta _3})A_2^{K - \xi - 1}$ $\kappa _1^2{\vartheta _2}{\vartheta _5}{\vartheta _6}$ $- {\beta _7}K(K - 1)A_2^{K - 2}\displaystyle{{\kappa _1^2\vartheta _5^2\vartheta _6^2} \over {{{(1 + \kappa _1^2\vartheta _2^2)}^2}}} - {[\arctan ({\kappa _1}{\vartheta _2})]^{K - 1}} \displaystyle{{{\kappa _1}{\beta _7}K} \over {{{(1 + \kappa _1^2\vartheta _2^2)}^2}}}$ $({\dot \vartheta _5}{\vartheta _6} + {\vartheta _5}{\dot \vartheta _6})$ $+ {\kappa _1}A_2^{K - 1}\displaystyle{{{\kappa _1}{\beta _7}K} \over {{{(1 + \kappa _1^2\vartheta _2^2)}^2}}}{\vartheta _5}{\vartheta _6} - {\beta _7}A_2^K\ddot \rho (t)$ $- {\beta _7}KA_2^{K - 1}\displaystyle{{{\kappa _1}{\vartheta _5}{\vartheta _6}} \over {1 + \kappa _1^2\vartheta _2^2}}\dot \rho (t)$ with *A*_2_ = arctan(*κ*_1_*ϑ*_2_). According to above equations, ${\dot \Gamma _3}$ and ${\ddot \Gamma _3}$ are continuous and bounded meaning ${\dot \Gamma _3}$ is uniformly continuous.

## Supplemental Information

10.7717/peerj-cs.793/supp-1 Supplemental Information 1 Simulink File.

## Additional Information and Declarations

### Data Availability

The following information was supplied regarding data availability: The Simulink model is available as a Supplemental File.
