# Supplementary figures and images for: Asymptotic stabilization of underactuated surface vehicles with actuator saturation
Source: PeerJ Comput Sci. 2021 Nov 24;7:e793. doi: 10.7717/peerj-cs.793 (PMC8627234; doi:10.7717/peerj-cs.793)

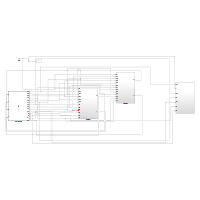

Supplement: Supplemental Information 1 [file peerj-cs-07-793-s001.slx › metadata/thumbnail.png]
